# Examining the Association of Grit Profiles With Big Five Personality and Achievement Among Iranian Foreign Language Learners

**DOI:** 10.3389/fpsyg.2021.801844

**Published:** 2021-12-24

**Authors:** Maryam Khodaverdian Dehkordi, Ali Akbar Jabbari, Golnar Mazdayasna

**Affiliations:** Department of English Language and Literature, Yazd University, Yazd, Iran

**Keywords:** grit, big five personality traits, L2 achievement, perseverance of effort, consistency of interest

## Abstract

Grit—trait-level perseverance and prolonged passion for primary goals—is related to several indicators of educational success such as academic achievement, engagement, and motivation. Although there is new evidence showing the beneficial effects of grit, most research has taken a variable-focused approach and consequently has failed to indicate how individuals with different grit profiles might have different academic results. The present study aimed to build on the existing literature on grit by investigating the relationship between grit, big five personality and L2 achievement. The participants of this study were 384 English as a Foreign Language (EFL) students from different academic majors studying at Yazd University. They had enrolled in General English (GE) courses. The results of the cluster analyses showed that there were three natural grit profiles (Cluster 1 = High Perseverance and High Consistency; Cluster 2 = High Perseverance and Low Consistency; Cluster = Low Perseverance and High Consistency) in the current study. The results of the multivariate analysis of variance (ANOVA) showed that students belonging to cluster 1 had the highest scores on big five personality traits and L2 achievement. The results also showed that when taking academic exams, high perseverance of effort and low consistency of interest were related to higher level of neuroticism and lower level of consciousness. Our study has some theoretical and practical implications. Regarding the theory, this study is related to the existing grit literature by elaborating the relationship between grit profiles, big five personality traits, and L2A. Regarding the practice, our findings affirm the significance of developing and implementing the big five personality traits interventions in grit prediction.

## Introduction

Both literally and figuratively, people take journeys throughout their lifetimes. Education life may be regarded as one of these journeys. In today’s world, whether it is in a formal or informal medium, people may start their education lives at the very early ages, and from that day forward, they try to achieve in the best way possible. In every aspect of life, to achieve success requires a set of skills, characteristics and personal traits that an individual must possess. However, regardless of the possession of specific factor having an impact on success, it is obvious that such characteristics or personal traits affect academic outcomes positively. Considering the fact that learning a language is like a marathon, not a sprint (Mercer, 2018), one of the personal traits that of vital importance is grit. Grit is as “a passion and perseverance to accomplish long-term goals whatever the obstacles and no matter how long it may take” (Duckworth et al., 2007, p. 541). The notion of grit refers to working tenaciously and passionately for a goal by preserving the struggle and interest for a long period of time; even if you confront failure, obstacles, and challenges during the whole process. Actually, this is what most people fail to do in case of experiencing failure at any point in their lives, and makes the all difference between successful people and other giving up easily.

Similarly, according to Mihaela (2015), one of the personal traits as that determines academic success is the personality factor. Therefore, this personality factor is a concern of education practitioners and researchers. One personality approach that can be used to identify aspects of personality is the big five personality approach (De Raad and Mlačić, 2015) and this approach is known as the structure of the personality model (De Raad and Mlačić, 2017). Neuroticism, extraversion, openness, agreeableness, and conscientiousness are 5 personality aspects of the big five personality approach model (McCrae and Costa, 2008). Regarding language learning, Rosito (2018) found that these five aspects of big five personality have a significant impact on the students’ academic achievement. While, Bhagat et al. (2019) came to the conclusion that personality is the combination of individual character as an asset for academic achievement. In short, big five personality can positively predict academic achievement.

Several research studies have been conducted on exploring the influences of grit on different educational settings and L2 achievement. However, little research has been conducted to investigate whether students who belong to different types of Big Five personality traits might differ in their L2 achievement and it is the principal goal of this research study. Studying the association between grit, big five personality and L2 achievement through cluster analysis can provide valuable information about the types of grit profiles may be associated with adaptive L2 achievement. The results may have an impact on the design of grit interventions for students with different grit profiles.

## Literature Review

### Grit and Achievement

Keegan (2017) asserted that grit as a non-cognitive trait significantly influence educational achievement and several research studies conducted on grit proposed that developing grit can help the students become higher-achieving learners. Therefore, exploring the characteristics of gritty students and the ways through which educators can help their students enhance their grit, can help the students become better achievers. According to Duckworth et al. (2007), two significant elements relating to grit are “consistency of interests,” which implies the preservation of the goals, interests or passions without letting them to change and “perseverance of effort,” which means incessant impulse to work with the aim of reaching the goal. In the field of SLA, early research used the common grit scale to measure language learners’ grit. To begin with, Duckworth et al. (2007) observed that in terms of Ivy League GPA, success at the national spelling bee contest, and withdrawal from military training, grit is a predictor of educational attainment. Similarly, the study of Akos and Kretchmar (2017) with undergraduate students shows that total scores reported by the participants themselves are positively related with GPA. In other words, higher total scores refer to higher GPA scores for the participants. The positive relationship between grit and academic performance is also shown in a study carried out Korean context by Lee and Sohn (2017), underlining that the grittier students have higher grades. Besides, Wei et al. (2019) found that there is a positive but low correlation between secondary school students’ grit and their English language scores in China. Whereas, Yamashita (2018) found no correlation between grit and GPA of Japanese learners. In a way that, the POE dimension of grit negatively influenced learners’ GPA. It should be noted that all of these studies used the common grit scales developed by Duckworth et al. (2007); Duckworth and Quinn (2009) and assessed learners’ grit globally rather than locally and relative to the L2 learning context.

Research lends support to the idea that the big five personality traits have been identified as predictors of grit. Big five personality traits are composed of extraversion, agreeableness, conscientiousness, openness to experience, and neuroticism (Rimfeld et al., 2016). In this study, the applicability of grit to L2 learners’ big five personality traits, and L2 achievement is examined. So, the relationship between grit profiles, its big five personality traits predictors, and its L2 learning achievements are investigated.

### Big Five Personality Traits and Achievement

Individuals have different characteristics, such as personality qualities related to specific behaviors, cognitive, and emotional patterns (Hogan et al., 1996). McCrae and Costa (1987) asserted that personality has a five-factor structure including extraversion, agreeableness, conscientiousness, neuroticism, and openness to experience or intellect. The operationalization of these big-five personality traits includes grit tendencies. They are essential predictors of grit. McCrae and Costa (1987) used the big five personality traits as a model for exploring the relationship between personality and different academic behaviors (Poropat, 2009). Following the big five model, a descriptive framework has been pointed out for many empirical research studies on the characteristics of predicting success (McCrae and Costa, 1987; Tupes and Christal, 1992; Goldberg, 1993; John and Srivastava, 1999). Barrick and Mount (1991) in a meta-analysis showed that big five conscientiousness is more related to job performance than the other factors. The role of these factors in grit with a specific emphasis on L2 learning is outlined in the following text.

#### Conscientiousness

It is identified as being organized, disciplined, and achievement-oriented. Conscientious people are mostly dependable, responsible, and able to set their goals and then persist to meet them. Highly intelligent people have a positive attitude toward challenges and setbacks in language learning (Barrick and Mount, 1991) as opposed to those who are mainly narrow-minded (McCrae and Costa, 1987). Among the big-five factors, conscientiousness is more related to motivation especially effort and persistence (Chamorro-Premuzic and Furnham, 2003). Geisler-Brenstein et al. (1996) asserted that there is a direct and positive relationship between conscientiousness and systematic learning.

#### Openness to Experience

Openness to experience or intellect is displayed as a preference for novelty and variety along with strong intellectual curiosity. Many research studies (Geisler-Brenstein et al., 1996; Slaats et al., 1997) have indicated that there is a link between Openness to experience and elaborative learning, meaningful learning, and constructive learning methods (Busato et al., 1998).

#### Neuroticism

It is defined as the degree of impulse control, emotional stability, and anxiety. Eysenck (2013) asserted that neuroticism is related to the lack of effective cognitive skills. Norem and Cantor (1986) showed that neuroticism can pave the way for motivation and effort, though it is understood as a defensive force, so anxious individuals in confronting a failure or challenge do their best to pre-empt it. However, according to Matthews and Zeidner (2004), neuroticism has a negative effect rather than a positive one. Poor critical thinking skills, analytical skills, and conceptual understanding are related to neuroticism because it supports high-level cognitive functions. Entwistle (1988) found that highly neurotic individuals have surface learning, instead of meaningful learning.

#### Extraversion

It refers to a higher degree of sociability, talkativeness, and assertiveness. From one side, extroversion could promote social manners like peer learning. On the other side, extroverts are inclined to reach cognitive closure over soon so they act poorly in reflective problem solving (Matthews, 1997; see also Matthews and Zeidner, 2004). In addition, highly educated individuals have a great investment of resources in doing difficult tasks. So, those who are sociable, impulsive, and easily distracted find it difficult to regulate their time and energy on these tasks.

#### Agreeableness

It is identified as being cooperative, helpful, and sympathetic toward others. Some research studies have found that it is positively correlated with hard work and superficial learning (Slaats et al., 1997; Vermetten et al., 2001). Also, Vermetten et al. (2001) asserted that agreeableness enables individuals to regulate their learning with external demands. It shows that agreeableness includes compliance and cooperation. Agreeable people are imperturbable in learning (McCrae and Costa, 1987).

Generally, the theoretical justifications of conscientiousness, openness to experience, extraversion, and agreeableness connection to components of language learning and L2 achievement are more than the theoretical justifications of neuroticism.

Previous research studies have shown that L2 achievement was linked to higher levels of conscientiousness (Chamorro-Premuzic and Furnham, 2003), extroversion (Matthews, 1997), openness to experience (Busato et al., 1998), agreeableness (Vermetten et al., 2001) and lower level of neuroticism (Entwistle, 1988). Also, research studies that have correlated grit with the personality dimensions specified in the big five model show similar results, but there are some differences. In one of the research studies, Duckworth et al. (2007) found that grit and conscientiousness have a strong correlation (*r* = 0.77, *p* < 0.001) followed by neuroticism (*r* = −0.38, *p* < 0.001), agreeableness (*r* = 0.24, *p* < 0.001), extraversion (*r* = 0.22, *p* < 0.001) and openness to experience (*r* = 0.14, *p* < 0.001). In another study, Lin and Chang (2017) explored the connections between personality dimensions and grit in high school students. Similarly, the results of the latter study also showed that conscientiousness is the superior predictor of grit (β = 0.44, *p* < 0.001) followed by neuroticism (β = −0.17, *p* < 0.001), openness to experience (β = 0.13, *p* < 0.001) and agreeableness (β = 0.11, *p* < 0.001). Furthermore, the results of these research studies indicated that extraversion did not have a predictive effect on grit. Based on these research studies, it is plausible to assume that grit may be associated to increased levels of conscientiousness, extroversion, openness to experience, agreeableness, and decreased extent of neuroticism in the L2 learning context.

## Purpose of the Study

The present study aimed to investigate the relationship between grit, big five personality, and L2 achievement among Iranian foreign language learners. In particular, our study expanded the body of evidence about the influence of grit on L2 achievement by examining how grit profiles may relate to the big five personality traits (i.e., extraversion, agreeableness, conscientiousness, openness to experience, and neuroticism).

In this research we have formulated the following hypotheses:

H1: There are specific grit profiles among L2 learners.
H2: Both POE and COI are essential elements of L2 achievement.
H3: The cluster characterized by a high POE and a low COI will exhibit a high level of neuroticism and low levels of other factors in comparison with the cluster exhibits high POE and COI.

## Materials and Methods

### Design of the Study

This study adopted a quantitative approach to find the answers of the questions. Questionnaire data from 384 EFL students comprising students of various academic majors who attend General English (GE) courses, assessed their grit and a set of five hypothesized predictors. A cluster analysis of the data through Ward’s method applied in the identification of probable L2 learner archetypes, which can provide evidence for the existence of L2 domain-specific grit profiles. Using multivariate analysis of variance (ANOVA), we examined links among the five predictor variables, grit profiles, and L2 achievement (GPA).

### Participants

**FIGURE 1:**
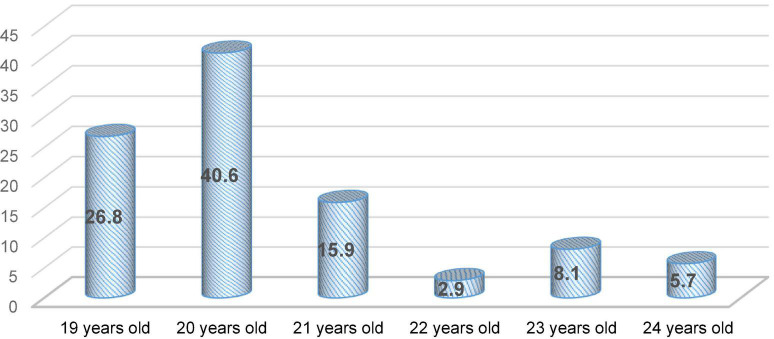
Distribution of the participants according to age status.

The current research was conducted at the Faculty of Humanities at Yazd University. Using random sampling, the participants were around 384 EFL students (60.4% male and 39.6% female) comprising students of various academic majors who attended General English (GE) courses. According to the Oxford Placement Test, their language proficiency ranged between lower-intermediate levels to upper-intermediate level. The participants of this study comprised students of different ages from 19 to 24. As can be seen in Figure 1, the highest frequency is related to the 20-year-old age group (40.6%) and the lowest frequency is related to the 22-year-old age group (2.9%).

### Instrumentation

#### Grit Questionnaire

The present study used the L2 short grit scale developed by Duckworth and Quinn (2009) to measure participants’ passion and perseverance for long-term goals. The items were rated on a five-point Likert scale from 1 (Not like me at all) to 5 (Very much like me). The 8-item grit scale has two 4-item subscales, namely perseverance of effort and consistency of interest which is presented in Supplementary Appendix A. The Cronbach’s alpha reliability coefficients of the perseverance and consistency subscales were 0.827 and 0.909, respectively.

#### Big Five Inventory

According to John and Srivastava (1999) the BFI consists of 44 short items, rated on a 5-point scale from strongly disagree to strongly agree. The BFI items are assigned to five measurement scales: Extraversion (E; 8 items), Agreeableness (A; 9 items), Conscientiousness (C; 9 items), Neuroticism (N; 8 items), and Openness to experience (O; 10 items).

### Data Collection Procedure

The data collection method was based on questionnaire. The short-term grit scale developed by Duckworth and Quinn (2009) was used to assess our participants’ level of grit (i.e., perseverance and passion for long-term goals). Besides, all participants were asked to fill Big Five Inventory (BFI; John and Srivastava, 1999), which is a 44-item questionnaire widely used to measure the big five personality factors of individuals. Participants rated items such as “I see myself as someone talkative” on a 5-point Likert scale with options ranging from (1 = disagree strongly) to (5 = agree strongly).

Since translation may jeopardize the reliability and validity of the instruments, a procedure called translation-back translation is performed to minimize this threat. Two non-affiliated researchers familiar with questionnaire construction translated the questionnaires into the participants’ L1 and these questionnaires were back-translated for consistency. Cronbach’s α was used to evaluate internal consistency. The Reliability of the scales is shown in Table 1. Following ethics approval, written consent forms from faculties in the universities and verbal participant assent were applied. They were told that their participation was entirely voluntary. The participants were assured about the confidentiality of their responses. The participants were asked to answer the Grit Scale and Five Big inventory and at the end of the semester, their L2 test scores as measures of their L2 learning achievement were collected.

**TABLE 1:** Reliability estimates of the variables.

Scales	Cronbach’s α	No of items in the final analysis
POE	0.827	4
COI	0.909	4
Conscientiousness	0.952	9
Neuroticism	0.964	9
Agreeableness	0.958	8
Extroversion	0.944	8
Openness to experience	0.967	10

*POE, perseverance of effort; COI, consistency of interest.*

At the end of the semester, participants’ final grades were obtained to assess their L2 achievement. They took a test that included reading comprehension, grammar, vocabulary, and pronunciation. In the Iranian educational system, scores range from 0 to 20 and the highest score is 20. If they get a minimum grade of 10, they will pass the course. Course grades are frequently used in L2 research (see Brown et al., 2018 for a review).

### Data Analysis

Confirmatory factor analysis was used to indicate the validity of the questions related to the research variables. According to CFA there was a significant correlation between the relevant latent variables and their corresponding indices. Ward’s method was used to provide cluster structure of the data and confirm H1. Through examining the agglomeration schedule three clusters were identified. Lastly, ANOVA was conducted in order to determine whether or not these clusters significantly differed on perseverance of effort and consistency of interests (H2 and H3).

## Results

### Descriptive Statistics and Latent Correlations Among Variables

Descriptive statistics and latent correlations can be seen in Table 2. As can be seen, the significance level of the correlation test between all research variables was less than 0.05. Therefore, it is perceived that there is a positive, direct and significant correlation between all the studied variables.

**TABLE 2:** Descriptive statistics and latent correlations among the measured variables.

Scales	*M*	SD	1	2	3	4	5	6	7
1. POE	3.427	0.855	1						
2. COI	3.440	0.901	0.813	1					
3. Conscientiousness	3.397	0.842	0.339	0.359	1				
4. Neuroticism	3.246	0.916	0.387	0.328	0.328	1			
5. Agreeableness	3.233	0.918	0.399	0.351	0.335	0.942	1		
6. Extroversion	3.386	0.830	0.290	0.235	0.231	0.333	0.340	1	
7. Openness to experience	3.240	0.921	0.392	0.339	0.329	0.953	0.948	0.322	1

### Confirmatory Factor Analysis of Research Variables

**FIGURE 2:**
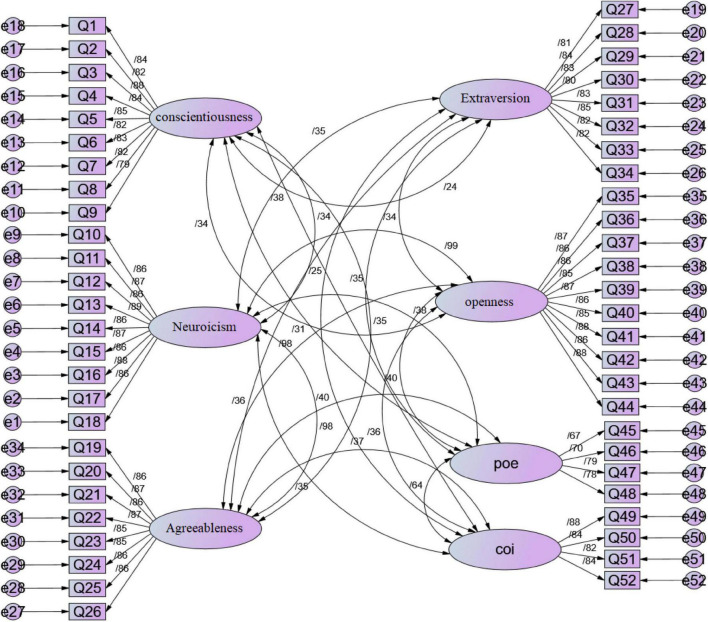
Standardized factor load coefficients of confirmatory factor analysis.

In this part of the research, the validity of the questions related to the research variables has been evaluated using confirmatory factor analysis. The standardized coefficient measurement model (Figure 2) shows that there is a significant correlation between the relevant latent variables and their corresponding indices.

### Grit Profiles

In order to identify different groupings of participants, based on their scores on two subscales of grit (i.e., POE and COI), a cluster analysis was launched. Although there are many ways to indicate the best number of profiles, it is more plausible to identify solutions with different numbers of profiles and finally indicate one that is more related to the analysis of the results and previous research (Marsh et al., 2009). To provide cluster structure of the data Ward’s method was used and through examining the agglomeration schedule the number of clusters was identified. Table 3 illustrates the three-cluster solution result in considerable changes in the agglomeration coefficients across all clusters. Also, prior empirical research on Chinese adolescents (Datu and Fong, 2018) suggested this three-profile solution.

**TABLE 3:** Grit cluster profile and their associations with big five personality factors.

	High POE		High POE		Low POE		

	**High COI**		**Low COI**		**Low COI**		
	***M***	**SD**	***M***	**SD**	***M***	**SD**	**Sig.**
POE	4.365	0.315	3.446	0.342	2.300	0.442	0.000
COI	4.373	0.310	2.831	0.703	2.202	0.422	0.000
L2A	18.384	1.067	17.173	1.025	14.660	1.577	0.000
Conscientiousness	3.693	0.832	3.115	0.863	2.943	0.909	0.000
Extraversion	3.647	0.776	3.363	0.817	3.118	0.831	0.000
Openness to experience	3.679	0.848	3.135	0.879	2.903	0.886	0.000
Agreeableness	3.692	0.848	3.113	0.985	2.896	0.850	0.000
Neuroticism	3.757	0.690	3.849	0.418	3.108	0.849	0.000

Following the previous line of research, the clusters in the study were named: (a) Cluster 1 (High Perseverance and High Consistency) = 117 students; (b) Cluster 2 (High Perseverance and Low Consistency) = 167 students; and (c) Cluster 3 (Low Perseverance and High Consistency) = 100 students. In particular, H1 was supported as the existence of specific grit profiles among L2 learners.

Analysis of variance was conducted in order to determine whether or not these clusters significantly differed on perseverance of effort and consistency of interests. As it can be seen in Table 4, these clusters significantly differed on POE at *p* < 0.05 level [*F*(2,381) = 868.735, *p* = 0.000]. Additionally, these clusters significantly differed on COI at *p* < 0.05 level [*F*(2,381) = 484.004, *p* = 0.000].

**TABLE 4:** Analysis of variance (ANOVA).

		Sum of squares	df	Mean square	*F*	Sig.
L2A	Between groups	772.089	2	386.044	265.926	0.000
	Within groups	553.096	381	1.452		
	Total	1325.185	383			
PE	Between groups	230.100	2	115.050	868.735	0.000
	Within groups	50.457	381	0.132		
	Total	280.557	383			
CI	Between groups	281.962	2	140.981	484.004	0.000
	Within groups	110.978	381	0.291		
	Total	392.940	383			
Neuroticism	Between groups	37.074	2	18.537	45.332	0.000
	Within groups	155.798	381	0.409		
	Total	192.872	383			
Conscientiousness	Between groups	35.397	2	17.699	23.579	0.000
	Within groups	285.988	381	0.751		
	Total	321.385	383			
Agreeableness	Between groups	38.397	2	19.198	25.678	0.000
	Within groups	284.857	381	0.748		
	Total	323.254	383			
Extraversion	Between groups	15.227	2	7.614	11.639	0.000
	Within groups	249.226	381	0.654		
	Total	264.453	383			
Openness to experience	Between groups	35.785	2	17.892	23.526	0.000
	Within groups	289.761	381	0.761		
	Total	325.546	383			
Grit	Between groups	142.644	2	71.322	406.053	0.000
	Within groups	66.921	381	0.176		
	Total	209.565	383			

Consequently, the results showed that the above-mentioned cluster profiles of grit differed in terms of big five personality traits: extraversion [*F*(2,381) = 11.639, *p* = 0.000], agreeableness[*F*(2,381) = 25.678, *p* = 0.000], conscientiousness[*F*(2,381) = 23.579, *p* = 0.000], openness to experience[*F*(2,381) = 23.526, *p* = 0.000], and neuroticism[*F*(2,381) = 45.332, *p* = 0.000].

In particular, H2 was supported as students belonging to cluster 1 had a higher POE (*M* = 4.365, SD = 0.315) and COI (*M* = 4.373, SD = 0.310) compared to students belonging to other clusters and consequently higher scores on L2A (*M* = 18.384, SD = 1.067). Conversely, students belonging to cluster 3 had a lower POE (*M* = 2.300, SD = 0.442) and COI (*M* = 2.202, SD = 0.422) and consequently lower scores on L2A (*M* = 14.660, SD = 1.577). It confirms that both POE and COI are essential elements of L2 achievement.

In terms of neuroticism, the findings showed that students in Cluster 2 outscored students in Cluster 1 and Cluster 3. H3 was fully supported because Cluster 2 had the highest level of neuroticism (*M* = 3.849, SD = 0.418) and lowest levels of other big five personality factors compared to the cluster 1.

## Discussion

In the current study, we examined the relationship between grit profiles, big five personality traits, and L2 achievement through cluster analysis. The findings showed three empirically based grit profiles: high perseverance and high consistency, high perseverance and low consistency, and low perseverance and low consistency. POE and COI were each positively associated with L2 achievement and are essential elements of L2 achievement; moreover, low COI will exhibit a high level of neuroticism and low levels of other factors of big five traits.

According to the three research hypotheses, there were three main findings to be discussed in this study. Firstly, the finding represented a three-profile solution of grit: profile 1 (high perseverance and high consistency), profile 2 (high perseverance and low consistency), and profile 3 (low perseverance and low consistency). This finding is an extension of previous research on grit profiles in Chinese adolescents (Datu and Fong, 2018). The present study adds a profile that is characterized by low perseverance and low consistency. This may indicate that language learners with low levels of determination to achieve success are also less likely to center personal interests and targeted tasks.

Secondly, we explored the moderating role of POE and COE in second language learners’ achievement. In line with prior research (Permzadian and Credé, 2016) the results showed that both factors of grit are essential elements of success in a way that perseverance of effort relates to the achievement of mastery in the face of failure, and consistency of interest is vital in involving in a task to accomplish mastery. The results supported H2 as the students belonging to the high perseverance and high consistency had the highest score on L2A than those belonging to low perseverance and low consistency. One possible interpretation is attributable to the Credé et al. (2017) which has emphasized a strong and direct association between grit, conscientiousness and academic performance. As such, students belonging to high POE and COI had the highest level of conscientiousness and then highest levels of POE, COI, and L2 achievement. Also, it confirms Barrick and Mount meta-analysis (1991) which has showed big five conscientiousness is more related to job performance than the other factors. While not explicitly aligned with research on grit, Yamashita’s (2018) on the relation between grit components and L2 achievement found no significant relation between leaners’ POE and their L2 achievement.

Thirdly, the current research showed that the association between high POE, low COI and L2 achievement was not significant for language learners. One possible explanation is that language learners with low level of COI are less likely to center personal interests and targeted tasks (Datu and Fong, 2018). As such, the low COI in language learners may have resulted in high level of neuroticism. Such an interpretation is in line with the current findings; that is, those belonging to high POE and low COI had high level of neuroticism and low levels of other factors in comparison with cluster 1. Our research confirmed what have been found in the existing literature on the negative effect of neuroticism on L2A (Matthews and Zeidner, 2004). Also, it confirms Entwistle’s (1988) that highly neuroticism individuals have surface learning, instead of meaningful learning.

Compared to the most positive factors for learners (conscientiousness, extraversion, and openness to experience, agreeableness), neuroticism may not be as prominent here. Our research results delineate that L2 learners who possess these constructs can elaborate high L2A despite a certain degree of neuroticism. Generally, the theoretical justifications of conscientiousness, openness to experience, extraversion, and agreeableness connection to components of language learning and L2 achievement are more than the theoretical justifications of neuroticism (McCrae and Costa, 1987). Despite the concentration on preventing or decreasing the negative constructs such as anxiety, hostility, depression, and impulsiveness that accompany L2 learning, it’s better to build up positive indicators (Oxford, 2016). Repositioning success in this way will be more effective because these positive indicators have successfully strengthened the learners’ L2 learning process and then help them successfully cope with and overcome daily stress and frustration. Given the point, that grit focuses on positive dealing with problems this may also make grit amenable to intervention at once. Our findings delineate that both POE and COI as two factors of grit have the potential to influence the key outcomes for instructed L2 learners.

Overall, this study shed light on L2 achievement through emphasizing the personality traits. Furthermore, we focus on the individual level of grit, irrespective of variable level. In fact, by a latent profile analysis we can generate evidence-based grit profiles for Iranian EFL learners. The aforementioned grit profiles in this study show that both POE and COI may cause similar developmental trend within each individual. In addition, although POE and COI are usually considered to be two positive personal attributes, they may also contain a negative aspect in a specific context as in cluster 2.

## Conclusion and Implications

By adopting a cluster analysis, the current study demonstrates when taking academic exams, high perseverance of effort and low consistency of interest were related to higher level of neuroticism and lower level of consciousness. Also, according to the present study all dimensions of big five personality; openness to experience, conscientiousness, extraversion, agreeableness, and neuroticism are a significant predictor of academic achievement whether negatively or positively. The results of this study can be applied by teachers, teacher trainers, and curriculum designers. A successful educational life is highly valuable and influential in a student’s future life. Therefore, different methods and techniques for enhancing students’ educational success should be emphasized and taught. Our study has some theoretical and practical implications. Regarding the theory, this study is related to the existing grit literature by elaborating the relationship between grit, big five personality traits, and L2A. Regarding the practice, our findings affirm the significance of developing and implementing the interventions of the big five personality traits in predicting grit.

## Limitation

For further research, some limitations of this research should be considered. First, students’ L2 achievement is measured through their final-term grades. Course grade is usually used to assess L2 achievement, but it has been criticized for validity issues (see Brown et al., 2018 for a review). So, we may have a more consistent measure of students’ L2 achievement by using standardized foreign language achievement tests. Second, only self-report questionnaires were used to evaluate grit and the big five factors. Some qualitative methods such as interviews can be used in future research investigate the related concepts. Finally, the findings of this study can only be extended to the participants of this study and other research should corroborate the results of this study.

## Data Availability Statement

The raw data supporting the conclusions of this article will be made available by the authors, without undue reservation.

## Ethics Statement

The studies involving human participants were reviewed and approved by the University of Yazd. Written informed consent to participate in this study was provided by the patient/participants in this study.

## Author Contributions

All authors listed have made a substantial, direct, and intellectual contribution to the work, and approved it for publication.

## Conflict of Interest

The authors declare that the research was conducted in the absence of any commercial or financial relationships that could be construed as a potential conflict of interest.

## Publisher’s Note

All claims expressed in this article are solely those of the authors and do not necessarily represent those of their affiliated organizations, or those of the publisher, the editors and the reviewers. Any product that may be evaluated in this article, or claim that may be made by its manufacturer, is not guaranteed or endorsed by the publisher.
