# Supplementary material for: Examining the Association of Grit Profiles With Big Five Personality and Achievement Among Iranian Foreign Language Learners
Source: Front Psychol. 2021 Dec 24;12:801844. doi: 10.3389/fpsyg.2021.801844 (PMC8739960; doi:10.3389/fpsyg.2021.801844)
Supplement: Supplementary file 1 [file Data_Sheet_1.pdf]

APPENDIX A
Short Grit Scale

Directions for taking the Grit Scale: Please respond to the following 8 items. Be honest – there are no right or wrong answers!

1. New ideas and projects sometimes distract me from previous ones.*
   - ❑ Very much like me
   - ❑ Mostly like me
   - ❑ Somewhat like me
   - ❑ Not much like me
   - ❑ Not like me at all

2. Setbacks don’t discourage me.
   - ❑ Very much like me
   - ❑ Mostly like me
   - ❑ Somewhat like me
   - ❑ Not much like me
   - ❑ Not like me at all

3. I have been obsessed with a certain idea or project for a short time but later lost interest.*
   - ❑ Very much like me
   - ❑ Mostly like me
   - ❑ Somewhat like me
   - ❑ Not much like me
   - ❑ Not like me at all

4. I am a hard worker.
   - ❑ Very much like me
   - ❑ Mostly like me
   - ❑ Somewhat like me
   - ❑ Not much like me
   - ❑ Not like me at all

5. I often set a goal but later choose to pursue a different one.*
    - ❑ Very much like me
    - ❑ Mostly like me
    - ❑ Somewhat like me
    - ❑ Not much like me
    - ❑ Not like me at all

6. I have difficulty maintaining my focus on projects that take more than a few months to complete.*
    - ❑ Very much like me
    - ❑ Mostly like me
    - ❑ Somewhat like me
    - ❑ Not much like me
    - ❑ Not like me at all

7. I finish whatever I begin.
    - ❑ Very much like me
    - ❑ Mostly like me
    - ❑ Somewhat like me
    - ❑ Not much like me
    - ❑ Not like me at all

8. I am diligent.
    - ❑ Very much like me
    - ❑ Mostly like me
    - ❑ Somewhat like me
    - ❑ Not much like me
    - ❑ Not like me at all
